# Knowledge, attitudes and practices on African tick bite fever of rural livestock communities living in a livestock-wildlife interface area in the Eastern Cape Province of South Africa

**DOI:** 10.1186/s12879-021-06174-9

**Published:** 2021-05-28

**Authors:** Tandiwe Katswara, Samson Mukaratirwa

**Affiliations:** 1 School of Life Sciences, Biological Sciences Section, College of Agriculture, Engineering and Science, University of KwaZulu-Natal, Westville Campus, Durban, 4000 South Africa; 2 One Health Center for Zoonoses and Tropical Veterinary Medicine, Ross University School of Veterinary Medicine, Basseterre, Saint Kitts and Nevis

**Keywords:** ATBF, Livestock-wildlife interface, South Africa

## Abstract

**Background:**

African tick bite fever (ATBF) caused by *Rickettsia africae* and transmitted by *Amblyomma* spp. ticks is one of the zoonotic tick-borne fevers from the spotted fever group (SFG) of rickettsiae, which is an emerging global health concern. There is paucity of information regarding the occurrence and awareness of the disease in endemic rural livestock farming communities living in livestock-wildlife interface areas in South Africa.

**Methods:**

The purpose of the study was to assess the level of knowledge, attitudes and practices on ticks and ATBF infection from a community living in livestock-wildlife interface areas in South Africa. A focus group discussion (FGD) was carried out followed by verbal administration of a standardized semi-structured questionnaire a month later to 38 rural livestock farmers (23 from Caquba area and 15 from Lucingweni area where *A. hebraeum* was absent). An FGD was conducted in Caquba (situated at the livestock-wildlife interface where *Amblyomma hebraeum* was prevalent on cattle and infected with *Rickettsia africae*) in the O.R. Tambo district of the Eastern Cape province of South Africa.

**Results:**

Results from the FGD and questionnaire survey showed that participants from the two rural communities were not aware of ATBF and were not aware that ticks are vectors of the disease. Respondents from Caquba reported of having frequent exposure to tick bites (91.3%, 21/23) specifically from the anthropophilic *A. hebrauem* which they were able to identify as *Qwelagqibe* in IsiXhosa (their vernacular). Thirteen out of 15 (86.7%) of respondents from Lucingweni reported that they had never been bitten by ticks, which corresponded with the absence of *A. hebraeum* from their locality as evidenced from results of a concurrent study on prevalence of ticks on livestock in the area. Both communities confirmed to being “very concerned” of tick bites and we presume this was more related to the localized wounds from the bites than to the diseases transmitted by the ticks.

**Conclusions:**

We recommend future studies encompassing seroprevalence of ATBF in Caquba and other communities at risk in South Africa including establishing surveillance systems to monitor the seasonal infection rates in ticks, cattle and humans.

**Supplementary Information:**

The online version contains supplementary material available at 10.1186/s12879-021-06174-9.

## Background

More than 100,000 cases of all reported zoonotic infections in the world are vector-borne, with spotted fever group (SFG) rickettsioses caused by species of the genus *Rickettsia* among the most widespread emerging and re-emerging zoonotic tick-borne infections [1]. Spotted fever group rickettsioses are associated with human infections, and ticks (Acari: Ixodidae and Argasidae) are the main vectors/reservoirs, and/or amplifiers. Ungulates play an integral role as primary hosts for the ticks while humans are accidental and dead-end hosts [2]. Humans are at risk for SFG rickettsiosis through exposure to bites from infected ticks during outdoor activities in rural or wilderness areas, and the epidemiology of each tick-borne rickettsial disease is reflected by the geographic distribution and seasonal activity of the tick vectors. Ungulates are primary hosts of infected ticks in the transmission of SFG rickettsiae, and several activities expose humans to tick bites with subsequent infection leading to tick bite fevers [3]. In addition, increased case reports of SFG rickettsioses especially African tick bite fever (ATBF) due to *Rickettsia africae* are recorded annually in travellers mainly from Europe, visiting game reserves and rural communities of sub-Saharan Africa [4].

Despite the abundance and wide distribution of the tick vectors, there is paucity of epidemiological information, limited diagnostic capacity and poor knowledge of the disease in rural communities of sub-Saharan Africa [5]. This paucity is because most tropical rickettsioses are often misdiagnosed as malaria, typhoid or acute febrile diseases. Until recently, Mediterranean spotted fever (MSF) caused by *R. conorii,* was the only SFG rickettsioses recognised in sub-Saharan Africa. However, due to the improvement in isolation methods and application of advanced molecular techniques, complemented with increasing medical awareness of tick-borne infections, several new SFG rickettsiosis which include ATBF have been identified [6]. Four tick-borne SFG *Rickettsia* spp. have been implicated as causes of human diseases in South Africa, namely; *R. africae* which causes ATBF, *R. conorii* which causes the MSF, *R. aeschlimannii* which causes innominate rickettsioses, and *R. sibirica mongolitimonae* which causes lymphangitis-associated rickettsiosis (LAR) [7–9]. ATBF is an emerging rural infectious rickettsiosis, and currently the most prevalent of these rickettsioses with many cases being reported annually from international travellers and tourists travelling from South Africa, Botswana and Zimbabwe [10, 11].

*Amblyomma variegatum* (tropical bont tick) is the main vector for ATBF throughout west, central, and eastern sub-Saharan Africa and *A. hebraeum* (South African bont tick), a tick of large ruminants and wildlife species, is the recognised vector and reservoir in southern Africa [12, 13]. A*mblyomma hebraeum*, the principal vector of ATBF in South Africa is a three-host tick, requires moisture, warmth and bush environments and do not survive in open grassland. The tick species is normally found at the coastal belts of South Africa from Port Elizabeth in the Eastern Cape province through KwaZulu-Natal and across Mpumalanga, Gauteng, Limpopo and North West provinces and mostly in areas where human and wildlife interface [14]. Surveys done indicate that ATBF is prevalent in South Africa and Zimbabwe and its pathogen, *R. africae*, has also been recovered from ticks in Ethiopia and Central Africa [15]. It has been shown that *R. africae* is transmitted trans-stadially and trans-ovarially in the host tick, hence may act both as reservoir and vector for *R. africae* [16].

In nature, vertebrates such as cattle are believed to be bacteremic for a very short period of time and hence considered as reservoirs of infection [17] resulting in classification of ATBF as zoonotic. A majority of population in South Africa live in rural areas and comprise of resource-poor livestock farmers who are regularly in contact with their livestock and thereby exposed to parasites and tick bites making transmission of zoonotic parasites possible [18].

Rural communities in southern Africa live in direct contact with their livestock and in some cases, in proximity to wildlife. Consequently, these communities are at high risk of being bitten by infected ticks from livestock and/or wildlife and getting infected by *R. africae*, transmitted by the anthropophilic *Amblyomma* spp. ticks. Despite several case reports of zoonotic tick-borne infections/fevers, especially from tourists from Europe and elsewhere visiting rural and game reserves in South Africa, there is dearth of information on the presence of the latter in populations at-risk in South Africa. The level of knowledge, attitudes and practices on the subject in these communities is also unknown.

The paucity of information regarding knowledge, attitudes and practices on the prevalence of ATBF, risk factors and the utilisation of appropriate prevention measures among rural communities and healthcare providers in Africa is demonstrated by some isolated studies carried out in Tanzania and Kenya [19, 20]. The studies depicted a low level of knowledge among rural livestock communities in SFG rickettsioses endemic areas and lack of effective prevention measures. Knowledge of community behaviours associated with risk of exposure to zoonotic tick-borne diseases is, therefore, critical. For the control and prevention of SFG rickettsioses in rural livestock communities in sub-Saharan Africa.

In view of the above, the aim of this study was to assess knowledge, attitudes and practices towards ATBF by rural livestock farmers in Caquba (living in a livestock-wildlife interface area) and Lucingweni (strictly livestock area) in OR Tambo district, in the Eastern Cape province of South Africa. We hypothesized that rural livestock farmers from Caquba living in a livestock-wildlife interface area where *A. hebraeum* and *R. africae* were endemic had different knowledge, attitude and practices on African tick bite fever than farmers from Lucingweni, living inland, in the Eastern Cape province of South Africa. This was achieved by conducting a focused group discussion (FGD) in Caquba, a rural livestock community at risk, to assess the level of knowledge from community members. This was followed by administration of a semi-structured questionnaire with questions informed by the result of the FGD in Caquba. Information on the prevalence of ticks in the two areas and whether they carried *R. africae*, the causative agent of ATBF, was obtained from a study which was concurrently conducted in the same study area (21) to corroborate information from the FGD and questionnaire interviews.

## Methods

### Study areas

The survey was conducted in the OR Tambo District in the Eastern Cape Province of South Africa. Two rural livestock communities, Lucingweni (King Sabata Dalindyebo local municipality, Mthatha) and Caquba (Port St Johns local municipality, Port St Johns) previously described [21] were selected for the survey. The two areas where the communities are located have contrasting environments, however, their socio-economic status is similar. Lucingweni is situated inland (latitude/longitude 31.459111/28.756333), with an altitude of 328.03 m above sea level. Caquba, on the other hand, is located close to the coast (latitude/longitude 31.6425/29.460028) with an altitude of 99.03 m above sea level.

Due to the contrasting landscapes, the two areas possess different grassland biomes where Lucingweni is sculptured with flat grounds, sparse and open grasslands in which *A. hebraeum* was presumed not to survive [22], although the species has recently been reported inland in cattle grazing in such environments [23], indicating a change in distribution likely due to climate change or animal movements. Caquba, on the other hand, is located at the livestock-wildlife interface and has a Savannah type of grassland consisting of medium to long grass, isolated bushes, and a few trees. This type of vegetation provides favourable conditions for the survival and breeding of *A. hebraeum*. Due to the contrasting landscape and vegetation in the two study communities, which influence the presence of the vector for ATBF, Lucingweni acted as the control community for the study and Caquba as the area of interest.

Both communities are communal lands dotted with rural villages and traditional homesteads all over the land but far apart from each other. Households in both communities rely on subsistence farming and/or extensive livestock rearing for their livelihood with a livestock capacity of approximately 300 cattle, sheep and goats per location. Most of the domestic ruminants are communally grazed and dipped weekly or bi-weekly for the control of ticks depending on the time of the year.

### Study design

The study was conducted concurrently with another study aimed to determine the prevalence of *A. hebraeum* in cattle and of *R. africae* in cattle blood and collected ticks. An exploratory mixed-method design was adopted to get an insight into the knowledge, attitudes and practices on ATBF amongst rural livestock farmers in Caquba (Port St Johns) and Lucingweni (Mthatha), in O.R. Tambo district in the Eastern Cape province of South Africa. The qualitative component of the study (FGD) preceded the quantitative component in order to increase the credence degree of the former [24] and also make use of the information from the FGD to formulate questions for inclusion in the subsequent questionnaire.

An FGD was conducted in Caquba to gather information on knowledge, attitudes and practices towards ATBF where the tick vector for ATBF; *A. hebraeum* and causative agent *R. africae* was found to be prevalent in a concurrent study. A cross-sectional survey using a standardized questionnaire which included both open- and closed-ended questions was carried out 30 days later in Caquba and Lucingweni. The questionnaire was pretested to five house-holds representatives outside our study areas to ensure correct understanding by the respondents. Data on the tick species collected from cattle and pastures from Lucingweni and Caquba, and the pathogens they harbour was obtained from a concurrent study during the study period [21].

### Data collection

Data was collected through FGD from participants living at the livestock-wildlife interface where *A. hebraeum* ticks infected with *R. africae* were prevalent in cattle in Caquba locality [21]. Findings from the FGD informed questions which were used in the subsequent semi-structured questionnaire on knowledge, attitude and practices on ATBF of livestock farmers in Caquba and Lucingweni. Inclusion criteria for participants of both the FGD and interviews were that participants should come from the study area and directly involved in livestock keeping, and/or actively involved in outdoor activities with a high risk of getting bitten by ticks and be 18 years of age or older.

The FGD was conducted by a social scientist with the assistance of a local translator and a note-taker. The discussion comprised of eight males and four female conveniently selected participants between the ages of 26 and 79 years, who were either stockmen or hunters who were actively involved in outdoor activities with a high risk of getting bitten by ticks. Themes of the discussion included knowledge on tick identification, ticks infecting humans and animals, diseases transmitted by ticks, their attitudes and practices towards ATBF if they mention it, and prevention and control measures they employ for ticks.

The cross-sectional quantitative survey was conducted 30 days later using a standardized semi-structured questionnaire (see Additional file 1) of which some of the questions were influenced by results from FGD. For example, the original questionnaire was modified using the information obtained from the FGD; like the common local name “*Qwelagqibe”* was used in the questionnaire instead of the descriptive term which we had for “*A. hebraeum*” and instead of mentioning ATBF as a specific disease we changed this to a general term of “tick-borne diseases”. Since the target population lived sparsely, and no prior data was available regarding the prevalence or occurrence of ATBF amongst livestock farmers, a standardized questionnaire with 31 open- and closed-ended questions translated into the local language (IsiXhosa) was verbally administered to 38 systematically selected households (23 from Caquba and 15 from Lucingweni). A household was defined as a housing unit with one or more persons accommodating it [19].

The questionnaire comprised of three thematic areas: demographic and putative risk factors, knowledge on ticks and tick-borne diseases, attitudes and practices on tick-borne disease risk, and utilization of commonly recommended tick-borne disease prevention behaviours. In addition to age, gender, and other demographic information, putative risk factors were obtained by asking participants to choose how often and for what length of time (less than 1 h, 2 to 4 h, all day) they engaged in outdoor activities such as farming/gardening, herding cattle and hunting in their communities. Participants were asked a series of questions of increasing specificity designed to elucidate their levels of knowledge and awareness about ticks and tick-borne diseases. Basic measures of knowledge included being able to identify ticks, knowing ticks could spread disease to humans, history of exposure to tick bites and treatment prescribed and having some awareness of ATBF or other tick-borne diseases. To ascertain attitudes and practices of risk, participants were asked if they were “very concerned”, “not very concerned”, or “not concerned at all” with being bitten by a tick and contracting tick-borne diseases. Finally, the questionnaire sought to determine the extent of preventive behaviours performed by respondents. Participants were asked whether they employed any measures to protect themselves from tick bites and the time of the year when ticks are abundant.

### Data analysis

The recording of the FGD was transcribed and, together with the notes, compiled during the discussion and used to extract the main themes on knowledge, attitudes and practices towards ticks and ATBF infections. Before data from the questionnaire was entered and analyzed, responses were checked for errors, completeness, and consistency. All data was first entered in MS Excel datasheets before being analysed using IBM Statistical Package for Social Sciences (SPSS), Statistical software version 25. Due to the nature of the questionnaire, not all questions were always answered by all respondents in the two communities and therefore, the n-values of the variables differed. Frequency distributions were used to describe the sample population, quantify knowledge, attitudes and practices towards tick-borne infections in man and livestock. Fisher test was used to compare knowledge, attitudes, and practices on ticks and ATBF in Caquba and Lucingweni. A resulting *p*-value < 0.05 was considered significance. The same procedure was applied to assess the association between the socio-demographic and household characteristics as putative risk factors for ATBF transmission across the study sites.

## Results

### Socio-demographic information for the FGD

A focus group discussion with 12 participants who had experience in livestock keeping was conducted in Caquba. The participants were a mixed group of eight males and four females, including the community head, housewives who owned livestock, retired mine and factory workers who reared livestock, and cattle herdsmen. Most of the respondents were born in the area and kept cattle during the pre- and post-South Africa independence era. Respondents were between the ages of 26 and 79 years, with most of them having attained primary education.

### Knowledge on ticks and the diseases they transmit/cause in man and livestock

Participants from the FGD conducted in Caquba mentioned and described the following tick species as common in their localities; *Rhipicephalus*, *Hyalomma* and *Amblyomma* ticks, which they said were more abundant during the rainy season than the dry season. All participants concurred that *A. hebraeum*, which they call *Qwelagqibe* in IsiXhosa, was the only tick linked with attacks to both man and livestock. They also reported of their experiences of having been bitten by the tick as confirmed below by one of the respondent. *“Qwelagqibe, a red coloured tick* (referring to the male *A. hebraeum*) *is the one that inflicts painful bites when one is bitten and causes bad sores in livestock”.* Respondent 1.

On further inquiry on where the ticks where found, one respondent said in the grass. The other respondents said the ticks were found on goats, cattle, pigs, and chickens, where they caused loss of blood and inflicted sores, especially in cattle. In humans, most of the respondents indicated that they were mostly attacked by ticks in parts of their bodies such as the ears, ankles or any soft tissue areas like the testes, causing itchy or swollen wounds. One respondent specifically said:

“*For me, I usually find the ticks attached around my testis”* Respondent 2

Although all the participants had been bitten by the *Qwelagqibe* tick (*A. hebraeum*), none of the participants knew that the tick is able to transmit disease(s) in humans and had never really paid attention to any symptoms such as fever, flu-like symptoms or muscle pain emerging after the tick bites except for the itchiness and sores at the bite site.

One of the respondents when asked said:

“*We are not aware that the Qwelagqibe ticks cause disease in us and have never noticed if we had a fever or fallen sick after the tick bites except for the itchiness and sores at the bite site*” Respondent 3

The stockmen exhibited some knowledge about the effects of ticks on their livestock and the common tick-borne disease that the majority knew was babesiosis/red water which they call *manzabovhu* in their local language. They knew the presenting clinical signs of the disease in cattle and the treatment prescribed. They also indicated that some of their cattle had died because of the disease:

“*Manzabovhu causes cattle to lose weight and the animal releases red, discontinuous urine. When the animal shows these signs, we go to the local veterinarian who gives us an injection containing Berenil/oxytetracycline as treatment”* Respondent 4

Besides babesiosis, the stockmen said the ticks also caused sores on the udder resulting in blockage of teats and reduced milk production in cows while in bulls, the ticks inflict sores on the prepuce rendering it useless for breeding purposes, leading to castration to resolve the damage.

### Attitudes and practices towards ticks and ATBF, prevention and control

Stockmen at the FGD believed that tick bites do not cause any diseases in man except for the localized wound caused by the tick bite. Whenever one was bitten by ticks, he or she would pull out the tick/s with their hands, then bath with an antiseptic like Dettol (chloroxylenol) or sulphur. They only applied an ointment bought from the pharmacy if a wound developed at the bite site. One of the respondents said:

‘*There are no measures or actions we take to protect ourselves from getting bitten by ticks. If one is bitten, they just pull out the tick and bath with Dettol or sulphur*’ Respondent 5

### Socio-demographic characteristics of the communities interviewed

A semi-structured interview questionnaire, with 31 questions translated into IsiXhosa was administered to 38 randomly selected respondents comprising of 23 livestock farmers from Caquba and 15 from Lucingweni. The socio-demographic data of the participants is summarised in Table 1. There was no difference in the number of males and females interviewed in the two localities although there were differences among age groups (*P* < 0.05). The level of education of participants was not the same (*P* < 0.05) in the two localities with more participants in Lucungweni having attained a minimum of secondary education.

**Table 1:** Socio-demographic characteristics of the study populations in Caquba and Lucingweni (OR Tambo District) in the Eastern cape of South Africa

.	Study areas		
	Caquba	Lucingweni	
Characteristic	N (%)	N (%)	***P***-value
^**#**^**Sex**			
Male	14 (60.9)	8 (53.3)	0.646
Female	9 (39.1)	7 (46.7)	
^**#**^**Age Group**			0.005*
18–25	0	5 (33.3)	
26–45	11 (47.8)	6 (40)	
46–65	8 (34.8)	4 (26.7)	
66 + years	4 (17.4)	0	
Average	48	33	
**Education Status**			0.005*
Primary and below	13 (39.1)	4 (26.7)	
Secondary and above	10 (26.1)	11 (73.3)	
^**#**^**Day to day activities**			0.968
Primarily outdoors	17 (73.9)	11 (73.3)	
Primarily indoors	6 (26.1)	4 (26.7)	
^**#**^**Livestock owned**			
Cattle	13 (56.5)	6 (40)	0.319
Goats	2 (8.7)	6 (40)	0.544
Sheep	8 (34.8)	8 (53.3)	0.002*
Chickens	17 (73.9)	10 (66.7)	0.632
Pigs	7 (30.4)	4 (26.7)	0.802
**Mean residency (years)**	47	17	
**Monthly income (ZAR)**			0.385
R0–450	14 (60.9)	7 (46.7)	
R460–850	4 (17.4)	6 (40)	
R860–1000	1 (4.3)	0	
> R1000	2 (8.7)	2 (13.3)	

Number sign (^#^) denotes risk factors that predispose individuals to tick bites and infection; ZAR = South African Rand; Asterisk (*) denotes significance at *P* < 0.05

Most of the respondents spent most of their time outdoors in both localities, with 74% (17/23) in Caquba and 73% (11/15) in Lucingweni spending their time outdoors. The outdoor activities they engaged in included working in the field, herding livestock and hunting. Fifty seven percent (13/23) of the households interviewed kept livestock in Caquba and 40% (6/15) in Lucingweni.

### Knowledge, attitudes and practices on ticks and ATBF

The most salient responses on knowledge, attitudes and practices towards ticks and ATBF infections in the two communities are shown in Table 2. There was a strong association between locality and being bitten by ticks as a larger proportion (91.3%; 22/23) of the respondents in Caquba were bitten by ticks compared to those in Lucingweni (13.3%; 2/15) (*P* < 0.001) (Table 3).

**Table 2:** Selected characteristics of knowledge, attitudes, and perceptions of livestock farmers towards ticks and tick-borne diseases of zoonotic importance in Caquba and Lucingweni areas of OR Tambo District in the Eastern Cape province of South Africa

Characteristics	N^**o**^. of respondents	Caquba (%)	Lucingweni (%)	***P***-value
**Ticks identified (ability to identify)**	**37**	**22**	**15**	
*Amblyomma hebraeum* (*Qwelagqibe*/ red) tick		22 (68.75)	0	< 0.001*
*Rhipicephalus* (Dark coloured/ umkhasi) tick		0	10 (31.25)	
Bitten by ticks	**37**	**22**	**15**	
Yes		21 (95.5)	2 (13.3)	< 0.001*
No		1 (4.5)	13 (86.7)	
**How the ticks were removed at the bite site**	**20**	**20**		
Plucked out		11 (47.8)	N/A	–
Crushed the tick(s)		5 (21.7)	N/A	
Used light from a matchstick		4 (17.4)	N/A	
**Treatment sought after a tick bite(s)**	**38**	**23**	**15**	
Health care facility		1 (4.35)	0	0.007*
Traditional healer		1 (4.35)	0	
Self-treatment		7 (30.4)	1 (6.7)	
No treatment		5 (21.7)	0	
No response		9 (39.1)	14 (93.3)	
**Perceived origins of ticks that bite humans**	**35**	**20**	**15**	
Domestic animals (Cattle, sheep, goats, cats, dogs and chicken/birds)		10 (43.5)	12 (66.7)	0.0436*
Humans		0	1 (6.7)	
Grass		1 (4.35)	0	
Do not know		9 (39.1)	2 (13.3)	
**Concerns about tick bites/tick-borne infection**	**36**	**21**	15	
Very concerned		19 (82.6)	13 (86.7)	0.559
Not very concerned		0	1 (6.7	
Not concerned at all		2 (8.7)	1 (6.7)	
**Individual protection from ticks**	**34**	**21**	**13**	
Bathed with antiseptic		2 (8.7)	0	
Wear protective clothing		0	6 (40)	
Do nothing to protect oneself		14 (60.9)	0	< 0.001*
Chemical control of ticks in livestock (Dip livestock and Use of traditional medicine)		3 (52.2)	1 (26.7)	
Environmental control (Avoid tick-infested animals/environment and Cut/mow grass)		2 (8.7)	6 (20)	

N/A denotes Not answered, Asterisk (*) denotes significance at *P* < 0.05

**Table 3:** Association of selected factors/characteristics of the respondents and being bitten by ticks in Caquba and Lucingweni in the Eastern Cape province of South Africa

Factor	Number of respondents	Bitten by ticks (%)	***P***-value
**Locality**			
Caquba	22	21 (95.5)	< 0.001*
Lucingweni	15	2 (13.3)	
**Sex**			
Male	21	15 (71.4)	0.183
Female	16	8 (50.0)	
**Age**			
18–25	5	1 (20.0)	0.082^a^
26–45	17	12 (70.6)	
46–65	11	6 (54.5)	
66 and above	4	4 (100)	
**Level of education**			
Primary and below	16	11 (68.8)	0.471
Secondary and above	21	12 (57.1)	
**Day to day activities**			
Primarily indoors	27	16 (59.3)	0.710^a^
Primarily outdoors	10	7 (70.0)	

^a^Fisher exact, Asterisk (*) denotes significance at *P* < 0.05

A significant association (*P* < 0.001) was also observed of tick species identified and locality with *A. hebraeum* (*Qwelagqibe*) correctly identified (68.7%; 22/32) by respondents from Caquba whilst 31.2% (10/32) respondents Lucingweni correctly identifying *Rhiphicephalus* (*Umkhasi*) ticks. Twenty-three participants out of the 38 interviewed from both areas admitted to being bitten by ticks at least once. In Caquba, 74% (17/23) of respondents also confirmed that their family members had at one point been bitten by ticks within the preceding 2 months and 30% (7/23) of them experienced more than one tick bite per week. The clinical signs mentioned by respondents from Caquba who were bitten by ticks ranged from flu-like (21%; 4/19) and malaria-like (32%; 6/19) and the remaining 47% (9/19) did not know either. When they were asked about tick-borne diseases of livestock, 61% (11/18) in Caquba mentioned babesiosis (redwater; *Manzabovhu*) as an example, but no one from Lucingweni had knowledge of any livestock disease transmitted by ticks.

Although 91% (21/23) of the respondents from Caquba said they had been bitten by ticks; 61% (14/23) of them did nothing to protect themselves from being bitten. There was a significant association (*P* < 0.05) in the choice of methods used by respondents for protection from tick bites with locality where in Caquba most respondents did nothing to protect themselves whilst in Lucingweni they either wore protective clothing or used environmental methods (Table 2). Interestingly, 46% (6/13) of the respondents from Lucingweni who were never bitten by ticks said they wore protective clothes such as overalls and gumboots when they are engaged in outdoor activities to prevent tick bites.

### Ticks identified from the study localities and the pathogens they harbour

To substantiate the information from the FGD and interview, information on the prevalence of ticks in cattle in the two rural communities of Caquba and Lucingweni and of *R. africae* in ticks and cattle blood was obtained from a concurrent study of the two areas [21]. *Amblyomma hebraeum* was the most prevalent tick in Caquba (46%; 233/504) and respondents were able to correctly identify the tick but was absent in Lucingweni. Of the *A. hebraeum* collected from cattle and pasture in Caquba, 55% (129/233) were positive for *R. africae*. *Rhipicephalus appendiculatus* and *R. (Boophilus) spp* were present in both Lucingweni and Caquba and were all negative for *R. africae* (Table 4).

**Table 4:** Prevalence of tick species form cattle and pasture and status of infection with *Rickettsia africae* in Caquba and Lucingweni localities in OR Tambo District, Eastern Cape province of South Africa

Tick species	Caquba		Lucingweni	
	Abundance(%)	Pathogen identified	Abundance(%)	Pathogen identified
*Amblyomma hebraeum*	233 (46.2)	*Rickettsia africae*	–	–
*Rhipicephalus (Boophilus)spp.*	182 (36.1)	–	191 (67.3)	–
*Rhipicephalus appendiculatus*	89 (17.7)	–	93 (32.7)	–

– = No *Rickettsia africae* identified

## Discussion

Ticks that infest livestock are often the reservoirs and/or vectors of zoonotic tick-borne diseases such as the emerging and re-emerging SFG rickettsioses such as ATBF [24]. The abundance of the tick vectors and the occurrence of ATBF can be attributed to the intensification of livestock farming in rural communities located at the livestock-wildlife interface. Thus, it is important to understand the level of knowledge, attitudes and practices towards such diseases by communities at risk by taking into consideration the social or contextual factors that influence disease transmission [25].

This study is among the few to be conducted with the aim of assessing knowledge, attitudes and practices on ATBF in a community living in a livestock-wildlife interface in eastern and southern Africa. Findings from this study showed that, although the respondents from Caquba (living in livestock-wildlife interface area) confirmed being frequently bitten by the anthropophilic *A. hebraeum* tick and were able to correctly identify the tick, they were not aware that the tick can transmit disease(s) to them, such as ATBF. Although they mentioned experiencing a variety of transient symptoms compatible with ATBF following tick bite, they did not consider them serious and did not relate these as due to a disease transmitted by ticks, but rather to the tissue damage caused by the bite from the tick. Only two respondents out of fifteen reported being bitten by ticks in Lucingweni where *A. hebraeum* was not found in their livestock. This finding is consistent with the established anthropophilic nature of *A. hebraeum* in Caquba, as a high number of respondents confirmed being bitten by the tick including their family members. In Caquba respondents had reasonable knowledge on tick-borne diseases transmitted to livestock whilst respondents from Lucingweni were unaware.

Despite a significant proportion of the respondents in Caquba having relevant knowledge about livestock tick-borne diseases (61%; 11/18) and experiencing frequent tick bites from *A. hebraeum*, they had no knowledge on ATBF. Our findings are consistent with other studies conducted by [26] Ndeereh et al. and [27] Mangesho et al. which cited that the local pastoralists in Tanzania and Kenya, respectively, had no knowledge about zoonotic tick-borne diseases although most of them expressed awareness and concern towards tick-borne diseases in livestock. We also confirmed the same with participants in the FGD, as they portrayed a picture of being more concerned about ticks and the damage they cause and diseases they transmit in livestock than on themselves. This can be accredited to the role of livestock, especially cattle, as an important source of livelihood and symbol of wealth in rural communities. Ndeereh et al. [26], however, attributed the pastoralists’ lack of familiarity with SFG rickettsioses in Kenya to the absence of a specific word for the disease in their local language or dialect. Although this could be true in the current study, unfortunately, the symptoms described by the few who responded could not be specifically pointed to those of ATBF beyond reasonable doubt.

Lack of knowledge on ATBF amongst respondents and preventive measures to protect oneself from tick bites, especially in Caquba where the tick vector (*A. hebraeum*) and the pathogen *(R. africae*) is prevalent, raises concerns about the potential risks posed by ATBF in the rural populace. Apart from the presence of *A. hebraeum* in Caquba, results obtained from a concurrent study on the eco-epidemiology of tick-borne rickettsial infections in the two areas of study showed that *Rhipicephalus appendiculatus* and *R. (Boophilus)spp* were present in both Lucingweni and Caquba and were negative for *R. africae* [21] and furthermore *Rhipicephalus* ticks have been reported to rarely bite humans [28]*.* The high prevalence of *A. hebraeum* infected with *R. africae* and detection of *R. africae* in blood from cattle in Caquba (21) made the community an area of interest in our study and suggested that ATBF could be circulating unnoticed in Caquba. Majority of respondents from Caquba did not mention/experience any symptoms after being bitten by *A. hebraeum*. This might have been due to respondents not being able to link the known symptoms of ATBF to tick bites due to lack of knowledge or that the community might have developed endemic stability to ATBF. Respondents from Caquba preferred self-treatment of tick bites which were based on home remedies. This practice corresponded with results from a similar study done on farmworkers from Malaysia [29].

Therefore, comprehensive educational and health campaigns for tick bite prevention among individuals in rural communities should include imparting knowledge about tick bites and ATBF, the presenting symptoms, as well as recommendations to seek medical attention once they develop symptoms after tick bites. This is pivotal, as studies done by Beaujean et al. [30] on public practices on tick-borne diseases have shown that ignorance on ability of ticks to transmit diseases, especially to humans, can make them underrate the consequences of being bitten by ticks.

The current study showed that respondents in Caquba, where *A. hebraeum* was prevalent, owned livestock, especially cattle and surveys done in West Africa have shown that seroprevalence of ATBF was high in communities where cattle farming coincided with the presence of the *Amblyomma* ticks [31]. Further studies to determine the seroprevalence of individuals at risk from Caquba locality for confirmation of this phenomenon is required. Some of the respondents in Caquba indicated that they spent more than 4 h in the fields and the whole day hunting in areas grazed by livestock without protective clothing, and according to Jensenius et al. [32], these outdoor activities predispose individuals to high risk of tick bites and subsequent infection.

Cross-tabulations of socio-demographic data of the two communities studied showed that there might be an association between factors such as age, gender, daily activities, livestock ownership, and the risk of being bitten by ticks. Although the seasonal activity of the ticks was not part of this study, responses obtained from the FGD and interviews showed that *A. hebraeum* was abundant during the rainy season which is usually from November to April and rainy season among other factors have been reported to be related to ATBF transmission [33, 34].

Education status could also be a potential risk factor to infection as alluded earlier by Beaujean et al. [30] who stated that lack of knowledge results in an underestimation of zoonotic tick-borne diseases and their implication. Since 39% (9/23) of the respondents in the study community of interest, Caquba, had only primary education, there is a likelihood that they might be unknowingly exposed to tick-bites thus placing themselves at risk of infection. Consequently, public health education campaigns are a prerequisite in mitigating potential zoonotic tick-borne infections in this rural livestock community and others at-risk in South Africa.

### Limitations of the study

The number of rural communities selected for this study and the number of questionnaires administered was constrained by the distances needed to be covered because of the remoteness of the study areas. In addition, the selection process for the FGD and semi-structured interviews might have be biased in that our study might have had participants with more knowledge or interest to participate. Furthermore, findings of this study may not be extrapolated to other provinces in South Africa because they are based on community participation reflecting the local preferences and priorities which may vary in different rural livestock communities.

## Conclusion

This study sought to assess the knowledge, attitudes and practices towards ATBF in selected rural livestock communities in the Eastern Cape province of South Africa. Respondents from both localities including Caquba where *R. africae* was detected in *A. hebraeum* ticks and cattle blood [21] and had frequent exposure to tick bites, were not aware of the existence of ATBF, although a few respondents mentioned having experienced symptoms related to the disease. The presence of the tick vector and cattle infected with *R. africae* in Caquba and its location at the livestock-wildlife interface makes it an area of interest for epidemiological studies and assessment of public health importance of ATBF to livestock farmers living in an endemic area.

Basic personal protection measures to avoid tick bites could be applied in areas where *Amblyomma* ticks are common together with application of chemicals insect repellents on skin and/or clothing [35, 36]. Our study showed that respondents had substantial knowledge on ticks and tick-borne disease of livestock and some of the measures they are currently applying are relevant in preventing tick bites and transmission of ATBF [37, 38]. Similar studies are recommended in other areas in South Africa where the *A. hebraeum* is prevalent, including seroprevalence studies of communities at risk, to close the epidemiological and awareness dearth in potential ATBF endemic areas.

## Supplementary Information

**Additional file 1.**

## Data Availability

The datasets used during the current study are available from the author on reasonable request.

## Abbreviations

ATBF: African tick bite fever
SFG: Spotted fever group
FGD: Focus group discussion
MSF: Mediterranean spotted fever
LAR: Lymphangitis-associated rickettsiosis
