# Supplementary material for: Knowledge, attitudes and practices on African tick bite fever of rural livestock communities living in a livestock-wildlife interface area in the Eastern Cape Province of South Africa
Source: BMC Infect Dis. 2021 May 28;21:497. doi: 10.1186/s12879-021-06174-9 (PMC8161941; doi:10.1186/s12879-021-06174-9)
Supplement: Supplementary file 1 — Additional file 1. [file 12879_2021_6174_MOESM1_ESM.docx]

# **Additional file 1**

# Community knowledge and perceptions on tick fevers and tick–borne zoonotic infection questionnaire

**SECTION A**

**Respondent demographic information**.

1. SEX: Male [ ] Female [ ]
2. AGE: ..................................
3. EDUCATION:

Primary [ ] Secondary[ ] Tertiary [ ] No education [ ]

1. DAY-TO-DAY ACTIVITIES:

Primarily indoors [ ] Primarily outdoors [ ]

1. RANGE OF ACTIVITIES:

Herding livestock [ ] Hunting [ ] Working in the fields [ ]

Others…………………

1. What is your monthly income?

R0-200 [ ] R210-450 [ ] R460- 650 [ ] R660-R850 [ ]

R850-R1000 [ ] <R1000 [ ]

1. How long have you been living in this area? ........................................
2. Do you own any livestock?

Cattle [ ] Sheep [ ] Goats [ ] Chickens [ ] Pigs [ ]

1. How do you manage your livestock?
2. Cattle

Communally grazed/free range [ ] Confined [ ] Mixed [ ]

1. Sheep and/or goats

Communally grazed/free range [ ] Confined [ ] Mixed [ ]

1. Chickens

Communally grazed/free range [ ] Confined [ ] Mixed [ ]

1. Pigs

Communally grazed/free range [ ] Confined [ ] Mixed [ ]

1. Who normally looks after your livestock?
2. Cattle

Yourself [ ] Farm worker [ ] Children/relative [ ]

1. Sheep and/or goats

Yourself [ ] Farm worker [ ] Children/relative [ ]

1. Chickens

Yourself [ ] Farm worker [ ] Children/relative [ ]

1. Pigs

Yourself [ ] Farm worker [ ] Children /relative [ ]

**SECTION B**

**Knowledge on ticks and tick-borne infections**

12. How often and for how long do you engage in outdoor activities such as:

(i) Gardening

>1Hr [ ] 2-4hrs [ ] All day [ ]

(ii) Cropping,

>1Hr [ ] 2-4hrs [ ] All day [ ]

(iii) Herding livestock

>1Hr [ ] 2-4hrs [ ] All day [ ]

(iv) Hunting

>1Hr [ ] 2-4hrs [ ] All day [ ]

13. Have you ever been bitten by a tick?

Yes [ ] No [ ] **Go to Q15**

14. IF YES, for how many times have been exposed to the tick bite (*Qwelagqibe*)?

>1tick bite/week [ ] tick bite within 2 months [ ]

1. Have any of your family members been exposed to tick bites?

Yes [ ] No [ ]

1. Can you please describe what the tick/s that bites you looked like?

...............................................................................................................

1. Local name for the tick/s …………………………………………..
2. How did you remove a tick when it had bitten you or a family member?

………………………………………………………………..

Use a tweezer [ ] crush the tick [ ]

Use a matchstick [ ] Apply petroleum jelly [ ]

1. Did you or the person who was bitten fall sick?

Yes [ ] No [ ]

1. What signs and symptoms did you or the person bitten present with?

……………………………………………………………………………

Don’t know [ ]

1. How long after being bitten did the person fall sick?

>12hrs [ ] 12-24hrs[ ] 1-2 days [ ] 2-5day [ ]

1 week +[ ] Other…………….

1. If you or any person(s) in your household fell sick after being bitten by a tick, where did the sick person seek treatment? (*tick* appropriate):

Health care facility [ ] Traditional healer [ ] Religious sect [ ]

Administered self-treatment [ ] Other [ ] (*specify*): ________________

1. Further to question 15, what was done for the sick person?

Nothing [ ] Received treatment: drugs [ ] herbs [ ]

Other ] (*specify)*: _______________

Hospitalized [ ] for how long: _______; Detained [ ] for how long: ________

1. What time of the year do you usually get tick bites?

Jan-Mar [ ] Apr-Jun [ ] Jul-Sept [ ] Oct-Dec [ ] Not sure [ ]

1. Where do the ticks that bite you come from?

Humans [ ] Cats and dogs [ ] Chickens/birds [ ]

Cattle [ ] Sheep and goats [ ] Not sure [ ]

1. During which season(s) are ticks in abundant?

………………………………………………………………

**SECTION C**

**Attitudes and perceptions on tick-borne disease risk and prevention measures**

1. Which diseases do the ticks cause in:
2. Humans

………………………………………………………………

1. Livestock

……………………………………………………………

1. Companion animals

…………………………………………………………………

1. Are you: (i) very concerned [ ]

(ii) not very concerned [ ]

(iii) not concerned at all [ ]

with being bitten by a tick or contracting a tick-borne disease?

1. How do you protect yourselves from getting bitten by ticks? (Give one most common method)

………………………………………………………………

1. How do you control or prevent ticks on your animals?

Dipping (specify ingredient and frequency)……….

Use of traditional medicine [ ]

1. How do you control ticks in the environment?

Use of acaricides [ ] Burn burrowing places [ ] Cut /mow grass[ ]

Other(specify)…………………………………….

**Thank you for your participation, we greatly appreciate your input**
